# Exploring the nature and variation of the stigma associated with loneliness

**DOI:** 10.1177/02654075221087190

**Published:** 2022-04-21

**Authors:** Manuela Barreto, Jolien van Breen, Christina Victor, Claudia Hammond, Alice Eccles, Matthew T Richins, Pamela Qualter

**Affiliations:** 1 Department of Psychology, 3286 University of Exeter, UK; 2 Department of Health Sciences, 3890 Brunel University, London, UK; 3 Radio Science Unit, British Broadcasting Corporation (BBC), London, UK; 4 Manchester Institute of Education, University of Manchester, UK

**Keywords:** Stigma, loneliness, loneliness stigma, age, gender, individualism, culture

## Abstract

The current study uses data from The British Broadcasting Corporation Loneliness Experiment to explore the social stigma of loneliness and how it varies by gender, age and cultural individualism. We examined stigmatizing judgements of people who are lonely (impressions of those who feel lonely and attributions for loneliness), perceived stigma in the community and self-stigma (shame for being lonely and inclination to conceal loneliness), while controlling for participants’ own feelings of loneliness. The scores on most measures fell near the mid-point of the scales, but stigmatizing perceptions depended on the measure of stigmatization that was used and on age, gender and country-level individualism. Multilevel analyses revealed that men had more stigmatizing perceptions, more perceived community stigma, but less self-stigma than women; young people had higher scores than older people on all indicators except for internal versus external attributions and people living in collectivist countries perceived loneliness as more controllable and perceived more stigma in the community than people living in individualistic countries. Finally, young men living in individualistic countries made the most internal (vs. external) attributions for loneliness. We discuss the implications of these findings for understandings of loneliness stigma and interventions to address loneliness.

In June 2019, the then UK Loneliness Minister Mims Davies launched a campaign with the explicit aim of tackling the stigma of loneliness (Abrams, 2019). As was the case with other campaigns around the world (e.g. in the US: ‘Far From Alone’ and ‘Commit to Connect’), this campaign reflects the recognition that the stigma of loneliness is problematic because it can (1) worsen the experience of being lonely and (2) make it harder to reach out to seek help, or to reconnect (Perlman & Peplau, 1981; Weiss, 1973). To address the stigma associated with loneliness, it seems crucial to understand what it might entail and how it might vary across socio-demographic characteristics. Since evidence addressing these issues is lacking, in the current paper, we use data from the British Broadcasting Corporation (BBC) Loneliness Experiment – including men and women aged 16–99 years, living in one of 237 countries, islands and territories – to examine a range of perceptions that are relevant to the understanding of the stigma associated with loneliness and explore how it might be shaped by gender, age and cultural individualism.

## What do we know about the stigma associated with loneliness?

Most definitions of loneliness converge on the idea that it is an aversive feeling that emerges when one’s social relationships are unsatisfying, in quality or quantity (Perlman & Peplau, 1981). A social stigma, in turn, a complex set of culturally shared beliefs that lead to the derogation and devaluation of specific attributes and discredit the individual bearing them (Goffman, 1963; Pescosolido & Martin, 2015). Social stigma is one way in which dominant members of society enforce their norms, ensuring people follow social norms and, if not, that they are excluded, or at least marginalized (Phelan et al., 2008). As such, the social stigma associated with loneliness corresponds to a constellation of beliefs that derogate and devalue those who feel lonely, so as to encourage them to have appropriate standards for social connection and to fulfil those standards.

Past research in this area has shown that people who feel lonely are often described in negative terms. For example, the few papers that examined this issue – in North America – revealed that those who feel lonely are perceived to be socially inept, poorly adjusted, unlikeable and generally incompetent (Borys & Perlman, 1985; Lau & Gruen, 1992; Tsai & Reis, 2009; Rotenberg et al., 1997; cf. Christensen & Kashy, 1998). A recent paper by Kerr and Stanley (2020) pointed out that past research in this area has typically confounded loneliness with poor social skills or behaviours, describing the people in the vignettes as both lonely and socially inept (e.g. Lau & Gruen, 1992). Kerr and Stanley (2020) argued that this is problematic because, though scholars used to believe that loneliness was the result of poor social skills and a preference for being alone (Jones et al., 1982), this is not supported by evidence. Indeed, people who report loneliness do not show a preference for being alone in their daily life (Queen et al., 2014), their social skills are at least as good as those of people who are not lonely (Gardner et al., 2005; Qualter et al., 2015), and they are sometimes even perceived as more friendly than non-lonely people (Christensen & Kashy, 1998; cf. Tsai & Reis, 2009). Crucially, Kerr and Stanley (2020)–also using a North American sample – manipulated loneliness in the absence of this confound and showed derogation of people feeling lonely only by college students, but no such stigmatizing perceptions among a more diverse community sample.

## Going beyond impressions

Although negative impressions of individuals reporting loneliness are key to understanding loneliness stigma, stigma can be expressed and understood in other ways too. A core distinction in the stigma literature is that between *endorsed* stigma (one’s own stigmatizing views of an identity or attribute) and *perceived* stigma (i.e. the belief that a particular identity or attribute is stigmatized in society; e.g. Pescosolido & Martin, 2015). Impressions of those who feel lonely fall under the category of endorsed stigma. Other perceptions in this category would be internal and controllable *attributions* for loneliness (Crandall et al., 2001; Jones et al., 1984; Weiner, 1995). Though loneliness can be predicted by personality characteristics (which qualify as internal attributions), these associations tend to be of small to medium size (Bueckner, Maes et al., 2020). Loneliness is strongly predicted by social determinants, such as changes in social networks due to life transitions (Buecker, Denissen et al., 2020), life circumstances (e.g. living alone, caring for a family member), socio-economic status, ethnic minority status, experiences with bullying or discrimination, disability, unemployment and living in a deprived area (Lasgaard et al., 2016; Matthews et al., 2019; Priest et al., 2017) – all of which are largely external and uncontrollable. As such, making largely internal or controllable attributions for loneliness neglects the range of structural, environmental and cultural factors that drive feelings of loneliness (Batsleer et al., 2018; Matthews et al., 2019), which is stigmatizing and hinders appropriate targeting of social interventions.

In addition to stigma endorsement, to gain a more complete picture of the stigma associated with loneliness it is important to examine indicators of *perceived* stigma (Pescosolido & Martin, 2015). Participants in a study carried out in the UK expressed fear that if they were to come forward to seek help for their feelings of loneliness, they would be simply told to ‘pull themselves together’ (Co-op Foundation and the British Red Cross, 2016). Another study, also in the UK, found that 81% of the young people surveyed cited fear of other people’s reactions as a barrier to speaking about loneliness (Co-op Foundation, 2019). These examples show that *perceived community stigma* contributes to norm enforcement by affecting the individual’s behaviour.

Finally, the derogation of those who feel loneliness and or perceived community stigma enforces normative expectations about sociality by engendering *shame* in those who feel lonely, as well as by encouraging them *to conceal* from others that they feel lonely. In the UK, a report by the Mental Health Foundation (2010) showed that one third of the people surveyed said they would be embarrassed to say they felt lonely. Shame can even affect the extent to which people admit to feeling loneliness when asked in anonymous questionnaires. In fact, scores on quantitative measures of loneliness are significantly higher, especially for male respondents, when the questions do not directly refer to feeling lonely (Borys & Perlman, 1985).

In sum, to understand the stigma associated with loneliness, it is important to go beyond the examination of the impressions people form of those who feel lonely. We need to examine other ways in which stigma can be endorsed and expressed (attributions for loneliness), and how it can be perceived, that is, whether people perceive there to be a stigma associated with loneliness in their community, and the shame and inclination to conceal loneliness that this might engender.

## Predictors of the stigma of loneliness

Our goal, in this paper, is to explore how these stigma-related perceptions might be shaped by gender, age and cultural individualism. As detailed, stigmatizing judgements target those who are perceived to endorse counter-normative attributes or behaviours (Goffman, 1963). In this vein, the stigma associated with loneliness is expected to exist because feeling lonely, or admitting to feeling lonely, would run counter to specific cultural beliefs about what is normal, desirable or acceptable for a particular demographic group and in a particular context. Might a respondent’s gender, age or individualism affect these beliefs?

### How might gender affect the stigma of loneliness?

A recent meta-analysis indicates that, overall, men and women experience loneliness to a similar extent across the lifespan (Maes et al., 2019). As such, loneliness is no more descriptively normative for either gender group. On the one hand, gender stereotypes encourage women to particularly value social connections, as well as to be well connected, which might motivate them to derogate lonely people to a greater extent than men, as well as to self-stigmatize more than men when feeling lonely. However, by encouraging men to care less about being socially connected, and generally to be more controlled and less emotional, gender stereotypes can motivate them to derogate lonely individuals more than do women, and to feel more shame when they feel the (for them counter normative) pain of disconnection. Evidence in this area is scarce and inconclusive. Classic research suggested that loneliness is more stigmatized by women than by men (Borys & Perlman, 1985; Lau & Gruen, 1992; Rotenberg & Kmill, 1992), but Kerr and Stanley’s (2020) study found no effect of gender on stigmatizing perceptions.

### Does age affect loneliness stigma?

To our knowledge, no studies to date have examined how age might affect loneliness stigma. Research before the COVID-19 pandemic (when this study was conducted) showed that loneliness is most prevalent precisely among young people (Barreto et al., 2020; Office for National Statistics, 2018; Schultz & Moore, 1988) and, in some studies, again in older age (Lasgaard et al., 2016; Luhmann & Hawkley, 2016; Victor & Yang, 2012; cf. meta-analysis by Mund et al., 2020). If so, one could argue that loneliness is more normative, and therefore less stigmatizing, among younger than older people. However, it is important to note that feelings of loneliness often remain hidden and might therefore not affect descriptive norms in such a direct way. Media portrayals before the COVID-19 pandemic tended to focus more on loneliness among elderly people, potentially contributing to normalizing it more for this age group. In fact, loneliness is often (wrongly) expected to be characteristic of older people (Pikhartova et al., 2016), whereas younger people are assumed to be the embodiment of sociality. Feeling lonely would make young people different from this perceived norm, so one could expect that feeling lonely is less unexpected and potentially less stigmatizing for older than younger people.

### How might loneliness stigma be affected by individualism?

So far research on the stigma associated with loneliness is restricted to North American contexts, but since any type of social stigma is inherently cultural (Link & Phelan, 2001), the stigma associated with loneliness might vary across cultures. In this paper, we focus specifically on the role of cultural individualism (vs. collectivism), or the extent to which a given society promotes independence and separateness versus interdependence and social connection (Hofstede, 1991). This focus was chosen because of the intrinsic link between this cultural dimension and relational norms in a given society (Triandis, 1995).

Based on these conceptualizations, one could expect that cultural environments that value independence, autonomy and self-reliance (individualistic societies) might be associated with more loneliness stigma because feelings of loneliness imply a deep need for connection that runs counter to those values. For example, referring to the US, Professor of Psychiatry Jacqueline Olds said ‘there is a stigma about loneliness because our culture romanticizes self-reliance’ (Mental Health Foundation, 2010). However, the opposite is also possible: The greater importance of connection and lower tolerance for deviance generally found in more collectivist societies (Triandis, 1995) might make the stigma associated with loneliness stronger in collectivist countries. As Chinese anthropologist Fei (1992) stated, in China, the failure to be well connected to others is ‘to be less than human’.

In the absence of evidence for how individualism affects the stigma of loneliness, we might consider how it affects self-reported loneliness. However, findings in this area are inconsistent, with some reporting more loneliness in collectivist environments and others reporting more loneliness in individualistic contexts (see Barreto et al., 2020; Heu et al., 2020). Therefore, it is unclear whether loneliness is more descriptively normative – and therefore potentially less stigmatized and stigmatizing – in more or in less individualistic societies. In addition, evidence for how cultural individualism affects self-reported loneliness might not say much about actual loneliness experiences because individualism might affect how people *actually* feel, but also (or instead) whether or not people *admit to* feeling lonely – a social desirability bias. That is, people might at the same time feel more lonely and more constrained by stigma in a given society, a combination that could misleadingly reveal lower levels of loneliness precisely where it is most felt.

## Overview of the study

This exploratory study aims to examine how multiple perceptions that shed light on the stigma associated with loneliness are patterned by gender, age and cultural individualism (vs. collectivism). We focus on the extent to which these factors predict who endorses most stigmatizing views (as indexed by impressions of those who feel lonely and attributions for loneliness), and who perceives and feels most stigma (as indexed by perceived community stigma, shame and inclination to reveal or conceal loneliness). The size and scope of the BBC Loneliness data allows us not only to examine effects of age, gender and cultural individualism with confidence, but also to examine the interactions between these factors. In addition, by including participants from 16-99 years old, living in a variety of countries, this sample allows us to address a major drawback of prior studies on this topic that focused uniquely on US populations, predominately of college students. This study was largely exploratory, so predictions for this study were not raised or pre-registered.

## Method

### Participants

The sample included 45,548 participants who described themselves as either women or men, lived in a country that appeared on the Hofstede database and provided input on their age (see Supplementary Materials for details). We only included participants who described themselves as men or women because our focus was on how gender is linked to social expectations that might make men and women differently vulnerable to the stigma associated with loneliness. This sample had a mean age of 50 years old (*SD* = 15.5 years), including 30,998 women (68%). Although most of the participants lived in the UK (*N* = 33,304, 73%), the effects of individualism (vs. collectivism) are produced by *variance* in individualism between countries (and so cannot be driven by one country specifically). Further demographic details of this overall sample are provided in Table 1, right column, with specific n per country provided in the supplementary materials. We did not ask participants what city they lived in, their race or ethnicity, whether or not they had a disability, or their educational status.

The survey was subdivided into branches to minimize the time it took to complete. The dependent variables causal attributions, controllability and participants’ own feelings of loneliness were part of the general branch of the survey – analyses for these variables focus on the total N of 45,548. The other dependent variables of interest here (impressions of people who feel lonely, community stigma, shame and inclination to conceal) were in the ‘stigma branch’ of the survey – analyses for these variables focus on the *N* = 9554 that took part in the stigma branch. Demographic information for this subsample is provided in Table 1, left column – the composition of this subsample was very similar to that of the overall sample.

**Table 1.** Characteristics of the sample used in the current study.

General	Stigma branch	Full survey
% women	68%	67%
% men	31%	32%
% other gender (excluded from analysis)	0.004%	0.005%
% prefer not to disclose gender (excluded from analysis)	0.004%	0.005%
Mean age in years (SD)	50.5 (15.4)	50.0 (15.5)
Median age in years	53	52
Age range in years	16–94	16–101
% residing in the UK	74%	73%
Mean Hofstede Individualism Index (SD)^a^	84.64 (13.70)	84.54 (13.80)
% falling below 3SD on Hofstede Individualism Index	4%	4%
Employment status^b^		
In full-time work	1858 (19%)	8797 (19%)
In part-time work	748 (8%)	3425 (7.5%)
In unpaid work	803 (8%)	3699 (8%)
Student (full or part-time)	869 (9%)	4244 (9%)
Retired	4088 (43%)	19,974 (44%)
Unemployed	2351 (25%)	10,800 (24%)
Socio-economic status		
Agreed that financial resources met their needs *very well*	3345 (35%)	15,218 (33%)
Agreed that financial resources met their needs *fairly well*	4662 (50%)	22,630 (50%)
Agreed that financial resources met their needs *poorly*	1540 (16%)	7560 (17%)
Mean self-reported social status (SD)–max. 9	5.38 (1.45)	5.36 (1.45)
Sexual orientation^c^		
% exclusively heterosexual	77%	76%
% predominantly heterosexual	12%	12%
% equally heterosexual and homosexual	2%	2%
% predominantly homosexual	2%	2%
% exclusively homosexual	3%	3.5%
% asexual	3%	3%
Romantic relationship status		
% single	28%	28%
% married or in civil partnership	31%	31%
% in a relationship, but not cohabiting	5.5%	5.5%
% separated or divorced	19%	19%
% widowed	6%	6%
Living situation		
% lives alone	41%	42%

*Note.* Percentages may not add to 100% due to missing responses and rounding.

^a^See Supplementary Materials for n and Hofstede Index per country.

^b^For employment status, participants could choose multiple options. The percentages reflect the percentage of the total sample who listed this option amongst their answers. As such, the percentages do not add to 100%.

^c^Measured using an adaptation of the Kinsey scale, retaining original wording.

### Dependent measures

#### Own loneliness

In our analysis of the stigma associated with loneliness, we include people’s own feelings of loneliness as a covariate.^1^ In doing so, we take into account existing evidence that loneliness affects self and other perceptions – with people who feel lonely being less positive about the self and their friends, but more positive about new contacts, than people who do not feel lonely (Christensen & Kashy, 1998; Duck et al., 1994; Jones et al., 1983; Tsai & Reis, 2009; cf. Kerr & Stanley, 2020; Rotenberg & Kmill, 1992). Felt loneliness is also associated with fear of rejection (Cacioppo et al., 2006; Watson & Nesdale, 2012), which might affect shame or inclination to conceal. Felt loneliness was operationalized by four questions from the UCLA loneliness scale that did not mention loneliness explicitly (Do you feel a lack of companionship? Do you feel left out? Do you feel isolated from others? Do you feel in tune with people around you? reversed, α = 0.84) with answers provided on five-point Likert-type scales from 1 (never) to 5 (always). For further details on how gender, age and country-level individualism predict feelings of loneliness, refer to (Barreto et al., 2020).

#### Impressions of people who feel lonely

The impression measure was based on Lau and Gruen (1992). Participants were presented with 21 semantic differentials on a scale of 1–7, with the positive trait on the left/lower end of the scale and its negative opposite on the right/higher end of the scale (e.g. Relaxed–Nervous). Higher scores on this measure reflect more negative impressions of people who are feeling lonely. Participants were asked to imagine ‘a person who is feeling lonely’ and to indicate how they viewed this person for each trait. Lau and Gruen (1992) differentiated four categories of Adjustment, Sociability, Competence and General Evaluation. However, in our dataset, combining all items generated a reliable scale (α = 0.91) and therefore, for the sake of parsimony, we analyse this measure as a single scale, with higher scores revealing more negative perceptions. Note that participants did not compare their impressions of ‘lonely versus non-lonely’ targets – therefore, stigmatizing impressions need to be inferred from the magnitude of absolute scores (i.e. whether or not these are above the scale mid-point). Results for the separate impression categories are given in the supplementary materials.

#### Causal attributions for loneliness

We had two measures of attributions of loneliness. First, we used a measure of attributions for another person’s loneliness developed for this study based on the work of Michela et al. (1982). These authors described a person who felt lonely due to either a lack of friends to do things with, or a lack of a boyfriend or a girlfriend, and asked participants to what extent each attribution was a likely cause of that person’s loneliness. Based on participants responses, the authors differentiated four categories of attributions: Internal and stable (e.g. ‘the person is afraid of being rejected’), internal and unstable (e.g. ‘the person doesn’t try hard enough’), external and stable (e.g. ‘The person believes other people […] aren’t interested in meeting new people’) and external and unstable (e.g. ‘there aren’t enough opportunities to meet people’). In our study we wanted to avoid pre-defining what had caused the person’s loneliness. To do so, participants saw statements that corresponded to each of these attributions and rated to what extent they thought the individual described in each statement felt lonely (e.g. ‘The person believes there is little chance of making a new friendship’; ratings from 1 = this person is not very lonely to 5 = this person is very lonely). High scores reflect a participant’s perception that particular behaviours cause loneliness and therefore reveal their endorsement for that particular attribution to loneliness.

Preliminary analysis of the causal attributions showed that, when grouping the attributions conceptually in line with Michela et al. (1982), reliabilities were low: None of the four subscales was reliable at the conventional level (α > 0.70). Focusing only on the Internal/External distinction yielded more acceptable reliabilities: The Internal factor reached satisfactory reliability (α = 0.72), while the External factor fell just short (α = 0.67). For this paper we, therefore, only differentiate between internal and external attributions, but even here the findings should be interpreted with caution. Note that we are not interested in whether people find each attribution plausible overall but, rather, in the tendencies to prefer one type of attribution over the other – with internal attributions reflecting more stigmatizing attitudes than external ones. Therefore, we created a difference score, whereby scores higher than zero reflect a tendency to prefer internal attributions for loneliness over external attributions (and therefore more stigmatizing attributions).

The second measure of attributions referred to perceived controllability of loneliness. We used four items developed for the purpose of this study (α = 0.83). Two items asked about the extent to which participants felt they could control *their own feelings* of loneliness: ‘If you think about when you feel lonely, to what extent do you agree or disagree that the feeling of loneliness is caused by…' (1) ‘something you can change?' (2) ‘something you can control?’ The other two items asked about the extent to which participants thought that *other people*, more generally, are able to control their feelings of loneliness: ‘When other people feel lonely, to what extent do you agree or disagree that the feeling of loneliness is caused by …' (3) ‘something they can change?' (4) ‘something they can control?' Participants indicated their agreement with these items on a scale of 1 (Strongly disagree) to 5 (Strongly Agree), so higher scores reveal more controllability perceptions and scores above 3 (mid-point) suggest more stigmatizing perceptions.

#### Perceived stigma in the community

This measure consisted of four items developed for this study and closely based on the Public subscale of the measure of Collective Self-Esteem (Luhtanen & Crocker, 1992). Items included, for instance, ‘In general, people in the community where I live tend to think that being lonely is a sign of weakness’. The items formed a reliable scale (α = 0.77) and were rated on a scale of 1 (Strongly disagree) to 7 (Strongly agree), with higher scores reflecting greater perceptions of stigma in the community and scores above 4 indicating more perceived community stigma.

#### Shame surrounding loneliness

We assessed the extent to which participants felt ashamed about feelings of loneliness, using three items developed for this study: ‘When I feel lonely, I feel ashamed about it’, ‘When I feel lonely, I am too embarrassed to admit it to others’, and ‘When I feel lonely, I don’t talk to others about it’. These three items were rated on a scale of 1 (Strongly disagree) to 7 (Strongly agree) and together, they formed a reliable scale (α = 0.80) with higher scores revealing more shame and scores above 4 revealing more felt stigma.

#### Concealing loneliness

We included a single item to examine inclination to conceal loneliness. Participants were asked to imagine they found themselves having a conversation with co-workers where the topic of loneliness came up. They were then asked whether, if they found themselves in this situation, they would reveal their own feelings of loneliness as part of that conversation. This item was rated on a scale of 1 (Would definitely reveal) to 7 (Would definitely not reveal), with a higher score on this item reflecting a greater inclination to conceal feelings of loneliness and scores above 4 suggesting more felt stigma.

### Procedure

Participants took part in an online survey launched in February 2018 on BBC Radio 4 and BBC World Service, and covered by several other media outlets. Data were collected during a 4-month period to increase the opportunity for a diverse to set of participants to take part. Participants accessed the study online and were first provided with information about the study. Those who agreed to participate answered a range of questions about their social life and their experiences with loneliness and were then randomly assigned to four different branches of the survey. The overall questionnaire included measures that did not pertain to the stigma of loneliness and that can be perused, along with the data and analyses scripts for this paper, here: https://osf.io/hv7t2.

Ethical approval was obtained for this study, prior to data collection, from the University Research Ethics Committee at the University of Manchester. The study followed ethical guidelines by the British Psychological Society and the Declaration of Helsinki (2013). The study took approximately 45 mins to complete. Those who participated did so on a voluntary basis.

### Analytical plan

We analysed how gender, age and country-level individualism (predictors) affected a series of stigma-related perceptions (dependent variables). Our data have a nested structure, since participants are nested within countries. We explore individual-level (age, gender) as well as country-level effects (individualism) on stigma surrounding loneliness. We analysed the data using the package *lmer* in R, creating a multilevel mixed effects model in which country of residence is the superordinate (level 2) factor. Specifically, country of residence is included as a random intercept. This model postulates that participants from the same country are more similar in their scores on the DV than participants from different countries. If the factor ‘Country’ reaches significance, this indicates that a multilevel structure is appropriate for the DV in question. As described above, we include people’s own feelings of loneliness as a covariate among the fixed effects. Given the large sample, we decided to adopt a more stringent significance criterion of *p* < .01, rather than *p* < .05.

## Results

### Descriptive statistics

Table 2 provides descriptive information for the key variables. All means were significantly different from the mid-point of the scales, though some of these differences were very small. Overall, participants had only very slightly stigmatizing scores when compared to the mid-point of the respective scales. Participants (1) reported positive impressions of the lonely target, (2) indicated that loneliness is uncontrollable and (3) did not perceive much stigma in the community. On the other hand, participants: (4) Attributed loneliness more internally than externally, (5) reported shame when experiencing loneliness and (6) indicated a preference to conceal the loneliness they experienced. Table 3 displays the correlations between the different stigma indicators. Attributions did not significantly correlate with the other stigma indicators. Impressions of people who feel lonely and controllability of loneliness had only very small correlations with other variables and these were in the opposite direction of what would be expected – for example, more positive impressions of people who feel lonely were associated with more shame, and perceived controllability of loneliness was associated with less shame. By contrast, perceived stigma in the community, shame and concealment were all more substantially inter-related and in the expected direction – with more perceived stigma in the community being associated with more shame and inclination to conceal. The full statistics for each of the dependent variables described below are provided in the Supplementary Materials (Tables A-F).

**Table 2.** Means, standard deviations and correlations for all stigma indicators.

	Mean (mid-point of scale)	Deviation from mid-point of the scale	SD	N observations
Own loneliness (covar)	2.55 (3)	−0.45	1.12	40,474
Controllability	2.95 (3)	−0.05	0.76	40,143
Causal attributions	0.06 (0)	0.06	0.51	34,317
Stigma in the community	3.76 (4)	−0.24	1.25	8461
Shame	4.51 (4)	0.51	1.63	8826
Impressions	4.62 (4)	0.62	0.71	9456
Tendency to conceal	4.58 (4)	0.58	1.71	9572

Variables in the first three rows were presented to all participants, whereas variables in the last four rows were only presented to participants in the ‘stigma branch’ of the study. Sample sizes vary further due to missing data.

All means were significantly different from the mid-point of the scale (all *p*s < .001 in t-test).

**Table 3.** Correlations amongst the central variables.

	2	3	4	5	6	7
1. Own loneliness	0.02	0.07	−0.26**	0.31**	0.39**	0.17**
2. Impressions	1	0.02	0.03*	0.06**	0.15**	0.03*
3. Attributions (diff score)		1	−0.01	0	−0.01	0.03
4. Controllability			1	−0.06**	−0.12**	−0.06**
5. Stigma in community				1	0.3**	0.14**
6. Shame					1	0.35**
7. Intention to conceal						1

^a^Internal and external attributions were each rated on a scale of 1–5. When creating a difference score, this means that the scale ranges from −4 (M_external_ = 5, M_internal_ = 1) to 4 (M_external_ = 1, M_internal_ = 5), with 0 as the mid-point.

* *p* < .01, ** *p* < .001.

### Impressions of people who feel lonely

The multilevel model showed that the random effect reflecting the differences between countries reached significance – model fit improved when including the random intercept, χ^2^ (1) = 21.68, *p* < .001. Participants’ personal feelings of loneliness did not significantly affect impressions, β = 0.01, *F* (1, 8687) = 2.26, *p* = .133. Regarding the predictors of stigma, none of those reached significance at *p* < .01.

### Internal attributions for loneliness

The multilevel model showed that the random effect reflecting the differences between countries reached significance, χ^2^ (1) = 21.69, *p* < .001. The size of the difference in internal versus external attributions was further predicted by participants’ own feelings of loneliness, β = 0.04, *F* (1, 32012) = 206.78, *p* < .001, *CI*_lower_ = 0.035, *CI*_higher_ = 0.046, such that those who reported feeling more lonely showed a greater tendency to make internal (stigmatizing) attributions over external attributions for loneliness.

With regard to our central predictors, there were main effects of gender, β = −0.07, *F* (1, 31778) = 114.69, *p* < .001, *CI*_lower_ = −0.08, *CI*_higher_ = −0.05 and age, β = 0.03, *F* (1,30818)=169.93, *p* < .001, *CI*_lower_ = 0.02, *CI*_higher_ = 0.04. Further, the interaction between gender and age reached significance, *F* (1, 32003) = 23.82, *p* < .001, as did the 3-way interaction between gender, age and country-level individualism, *F* (1, 29886) = 6.44, *p* = .011, albeit slightly above our criterion of *p* = .01. No other effects were significant with *p* < .01.

**Figure 1.**
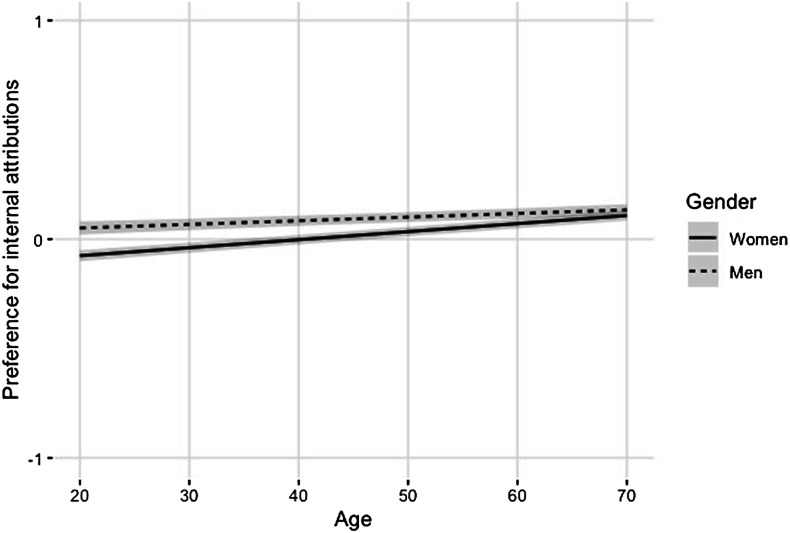
The interactive effect of gender and age on participants’ tendency to make interval versus external attributions for loneliness. *Note.* As can be seen in Table 2 above, the y-axis has a range of −4 to +4, but for the purposes of readability, the figure gives a restricted range.

Breakdown of the interactions showed that young women were the least likely of all groups to differentiate between internal and external attributions for loneliness. This effect is represented graphically in Figure 1. The significant 3-way interaction further indicated that these effects were more pronounced in more individualistic cultures: In highly individualistic cultures, young women indicated no preference for internal (vs. external) attributions (*M* = −0.03, *SD* = 0.70); this differentiates them from young men, *Μ*_*diff*_= −0.10, *t* (31804) = −8.54, *p* < .001, *CI*_lower_ = −0.12, *CI*_higher_ = −0.08, and from older women, β =0 .06, *t* (28621) = 12.41, *p* < .001, *CI*_lower_ = 0.05, *CI*_higher_ = 0.07, who showed a slight preference for internal (stigmatizing) attributions.

### Perceived controllability of loneliness

The multilevel model showed that the random effect reflecting the differences between countries reached significance, χ^2^ (1) = 303.80, *p* < .001. Perceived controllability of loneliness was also affected by felt loneliness, such that those who felt more lonely perceived loneliness as less controllable, β = −0.20, *F* (1, 37191) = 2839.39, *p* < .001.

Further, there were main effects of the three predictors of interest: gender (*M*_men_ = 3.04, *SD*_*men*_ = 0.87, *M*_women_ = 2.97, *SD*_*women*_ *=* 0.90), *M*_diff_ = 0.07, *F* (1, 37191) = 86.39, *p* < .001, *CI*_lower_ = 0.06, *CI*_higher_ = 0.09, age, β = −0.03, *F* (1, 37191) = 16.23, *p* < .001, *CI*_lower_ = −0.04, *CI*_higher_ = −0.01, and individualism, β = −0.02, *F* (1, 37191) = 14.32, *p* < .001, *CI*_lower_ = −0.04, *CI*_higher_ = −0.01. Older people, women and those in more individualistic cultures perceived loneliness as less controllable relative to younger people, men and those in collectivistic cultures. No other effects were significant with *p* < .01.

### Perceived stigma in the community

The multilevel model showed that the random effect reflecting the differences between countries reached significance, χ^2^ (10) = 47.68, *p* < .001. Perceived stigma in the community was predicted by feelings of loneliness, β= 0.37, *F* (1, 7843) = 773.78, *p* < .001, *CI*_lower_ = 0.34, *CI*_higher_ = 0.39, so that those who felt more lonely perceived more stigma in the community.

Further, there were main effects of the three predictors of interest: Gender, *M*_diff_ = −0.15, *F* (1, 7843) = 28.22, *p* < .001, *CI*_lower_ = −0.21, *CI*_higher_ = −0.10, age, β = − 0.18, *F* (1, 7843) = 161.94, *p* < .001, *CI*_lower_ = −0.23, *CI*_higher_ = −0.14, and individualism, β= −0.09, *F* (1, 7843) = 16.64, *p* < .001, *CI*_lower_ = −0.15, *CI*_higher_ = −0.03. Older people, women and those in more individualistic cultures perceived *less* stigma in the community relative to younger people, men and those in collectivistic cultures. No other effects were significant with *p* < .01.

### Shame surrounding loneliness

The multilevel model showed that the random effect reflecting the differences between countries reached significance, χ^2^ (1) = 47.31, *p* < .001. Shame surrounding loneliness was predicted by feelings of loneliness, β = 0.60, *F* (1, 8199) = 1362.53, *p* < .001, *CI*_lower_ = 0.57, *CI*_higher_ = 0.63, such that people who felt more lonely reported greater feelings of shame.

Further, shame surrounding loneliness was predicted by main effects of gender, *M*_diff_= 0.13, *F* (1, 8199) = 13.27, *p* < .001, *CI*_lower_ = 0.06, *CI*_higher_ = 0.20, and age, β = −0.29, *F* (1, 8199) = 226.24, *p* < .001, *CI*_lower_ = −0.34, *CI*_higher_ = −0.23. These main effects show that shame was higher amongst women and amongst younger people, relative to men and older people. No other effects were significant with *p* < .01.

### Inclination to conceal loneliness

The multilevel model showed that, in this case, the random factor Country did *not* impact model fit, χ^2^ (1) = 0.37, *p =* .545. Participants’ inclination to conceal loneliness was affected by their own feelings of loneliness, β = 0.27, *F* (1, 8802) = 236.76, *p* < .001, *CI*_lower_ = 0.24, *CI*_higher_ = 0.31, such that people who felt more lonely reported a greater tendency to conceal it.

In addition, inclination to conceal loneliness was predicted by a main effect of age, β = −.18, *F* (1, 8802) = 97.19, *p* < .001, *CI*_lower_ = −0.25, *CI*_higher_ = −0.12 – younger people were more inclined to conceal loneliness than older people. No other effects were significant with *p* < .01.

## Discussion

We explored the stigma-related perceptions associated with loneliness and how those vary across gender, age and cultural individualism. We distinguished between participants’ endorsement of stigma (i.e. their own stigmatizing attitudes towards those who feel lonely: impressions and attributions), and their perceived stigma (i.e. the extent to which they perceived a stigma associated with loneliness in their community, and their feelings and attitudes towards their own loneliness: shame and concealing). Overall, means were around scale mid-points, so there is little evidence of endorsement or perceived stigma in the overall sample. However, the extent to which stigma-related views were expressed was patterned by the independent variables.

### Effects of participants’ own loneliness

Participants’ own loneliness was significantly related to all stigma indicators except for impressions of people feeling lonely: The more participants felt lonely the more they made internal (vs. external) attributions for loneliness, the less they perceived loneliness to be controllable, the more they perceived loneliness as stigmatized in their community, the more shame they felt when feeling lonely and the more inclined they were to conceal their feelings of loneliness. These associations were not core to our focus, but they are nevertheless interesting and consistent with past research showing that loneliness is associated with fear of negative evaluation and fear of rejection (Cacioppo et al., 2006; Watson & Nesdale, 2012).

### Effects of participant gender

Like Kerr and Stanley (2020), we did not find any effect of gender (or indeed of any predictor) on impressions of loneliness. However, young male participants differentiated more between internal and external attributions for loneliness and male participants of all ages were more likely to see loneliness as controllable and to perceive a stigma around loneliness in their community. These findings suggest that loneliness is more stigmatized by men than by women, but also that men are more exposed to this type of stigma than women. At the same time, however, women were more likely than men to report that they would feel shame when feeling lonely. This later result might be less related to actual experiences of shame linked to loneliness and more to the phenomenon that men are less likely to express shame than women, stemming from differences in the extent to which men and women are socialized to feel or express shame (Else-Quest et al., 2012).

### Effects of participant age

Older people were more likely to make internal (vs. external) attributions for loneliness, but younger people were more likely to perceive loneliness as controllable. Though participants were asked to make attributions for another person’s loneliness, their answers might draw on differences in the predictors of loneliness across the lifespan and therefore in respondents’ own loneliness experiences. While both internal and external factors play a role across the lifespan, loneliness in older age is more strongly linked to factors such as health issues and widowhood, which are internal and uncontrollable, whereas in younger people loneliness is most often predicted by concerns about friendships, which are more ambiguously attributed (Qualter et al., 2015). Participants may have, therefore, projected from the causes of their own lonely feelings to how they perceived loneliness to emerge in others.

Younger people also perceived more stigma in the community, expressed more shame and greater inclination to conceal loneliness. This might reflect the (erroneous but prevalent) idea that loneliness is more prevalent among older people, which would by definition make loneliness more normative in older participants, and lead younger participants to feel more vulnerable to stigma from the community, more ashamed, and more keen to conceal their loneliness. In a nutshell, representing loneliness as a problem of old age is not helpful to young people as it makes it more deviant at their age.

### Effects of country-level individualism

Participants living in individualistic countries were more likely to make internal (vs. external) attributions for loneliness. The only significant interaction revealed in this study showed that internal attributions were prioritized over external attributions the most by older male respondents living in individualistic countries. However, the only two other measures that were affected by cultural individualism showed that it was those living in collectivist countries (i.e. low individualism) who were most likely to make controllable attributions for loneliness and to perceive stigma in their community. This might reflect the idea that interdependence is so core to collectivist cultures that being disconnected is perceived to be a deliberate choice (Fei, 1992). At the same time, collectivist cultures more tightly control individual behaviour, particularly with regard to social relationships (Triandis, 1995), and this control can only be effective if group members are aware that they will be incur social costs if they deviate from the norm, reflected in higher perceived community stigma.

### Bringing it all together

In sum, our results generally reveal that stigmatizing views of loneliness are relatively stronger amongst men, young people and people living in collectivist societies. These results can be explained by reference to the social norms those participants are expected to adhere to. Collectivist societies thrive on strong social networks and the stigma of loneliness might play an important role in encouraging people to remain well connected (Triandis, 1995). Sociality is also key in human development, particularly during adolescence and young adulthood (Qualter et al., 2015), so again the stigma associated with loneliness might play an important role in ensuring that happens. Effects of gender are less clear – stereotypes describe and prescribe more sociality for women than for men (Fiske et al., 2002), while, at the same time, men and women do no differ in actual social engagement or satisfaction with their social ties (Maes et al., 2019). However, in any society, men are expected to be less emotional than women, so the stigma associated with loneliness for men might refer more to a derogation of emotionality than to a derogation of social disconnection per se.

### The importance of different stigma indicators

The current study shows a complex and nuanced picture of how the stigma of loneliness is manifested, since different indicators revealed slightly different patterns of results. We proposed to go beyond people’s impressions of those who feel lonely, which is the way in which this has been addressed in the past. How useful was that? We found the measures of impressions and attributions related weakly with the other indicators, and in the opposite direction of what was expected. In particular, we had expected that greater perceived controllability of loneliness would be associated with greater stigma and shame, but found the opposite. When considering this result, it is important to note that the bivariate correlations did not include a multilevel structure. When Country was included as a higher-level factor, the negative relationship between shame and controllability no longer reached significance, suggesting that this negative relationship may be driven by extraneous differences between countries (for instance, the extent to which people value controllability, as suggested above). By contrast, perceived stigma in the community, shame and inclination to conceal correlated with each other in expected ways. Of course, the impression and attribution measures referred to other people, whereas the remaining measures referred to the self, which might explain these patterns of association. One might argue that perceived community stigma, shame and concealment are more relevant to understanding individual experiences with stigma, which in turn predict their behaviour – that is, whether or not they disclose feeling lonely, and whether or not they seek social support. In turn, impressions and attributions might be more relevant to whether or not such support is available. If so, then an understanding of the stigma associated with loneliness benefits from considering the multiple ways in which it can be expressed and experienced. These results, therefore, highlight the importance of examining a variety of stigma-related perceptions, so as to gain a more complete understanding of how it operates in a given context. Campaigns or interventions that address only one aspect of stigma might miss the way their particular target group experiences the stigma of loneliness, or address it incompletely.

### Limitations and Future Directions

It is, of course, important to acknowledge that this study has important limitations. A major limitation is that the study did not include a representative sample of residents of each country. This, together with the fact that the study was advertised primarily through the BBC radio channels (though it was also well covered in other media), might have skewed the sample towards older retired participants with a higher education level, who might be better informed about loneliness experiences and, therefore, stigmatize those less. Future research might wish to carry out similar analyses with representative samples. However, it is important to note that what we lost in representativeness of the population of each country, we gained in representativeness of the individualism-collectivism construct (usually represented in research by only a handful of countries, at best), since, with this method, we were able to collect data that spans the complete continuum specified by Hofstede.

Another limitation is that we did not use participants’ own definitions of loneliness, which might themselves vary by gender, age or individualism. Although a recent study has shown more variability in loneliness definitions within cultures than across cultures (Heu et al., 2021), it is, of course, possible that gender and age are two of the predictors of this within-culture variability. Future research might be able to examine this in more detail, as well as its implications for loneliness stigma. In addition, the study relied on self-reported measures that are vulnerable to socially desirable responses. This does not allow us to differentiate effects that pertain to how people actually feel from effects that reflect their willingness to abide by what they perceive to be normative local, age- or gender-appropriate normative standards. As such, it is important to regard our findings as what people say about their views on loneliness, rather than necessarily what they think. That said, this can be seen as having good external validity, since stigma tends to play out in public contexts, which is where social desirability is most salient. A more problematic measurement issue is that the measure of internal (vs. external) attributions was not highly reliable. This might explain why in some cases this measure revealed patterns different from the other measures (e.g. whereas young people scored higher than older people on all the other measures, older people made stronger internal vs. external attributions). Future research might wish to examine this further.

The large sample can easily lead to the detection of effects that are so small they might not be very meaningful. However, it is important to note that small effects obtained in such a diverse sample and under uncontrolled (or ‘noisy’) conditions can actually reflect larger effects in samples that are more homogeneous on variables that are not central to the analysis and obtained under more controlled conditions. We, therefore, take these effects seriously, while keeping in mind they need to be replicated with different methodologies. It is also true that participants were unequally distributed across country. However, multilevel analyses of country effects are sensitive primarily to the number of countries included (which in this study was the 101 that can be coded on the basis of Hofstede’s coding system), rather than to the number of participants per country. Still, future research might wish to examine this issue with more equally sized samples.

It is also important to acknowledge that participants voluntarily chose to participate in this study on loneliness, a framing that might have influenced the extent to which they stigmatized loneliness, or thought of loneliness as socially stigmatized. This could have improved attitudes and perceived stigma, implying that data collected in other circumstances might actually reveal more stigma than we found. However, it is unclear whether or why one would expect gender, age and cultural effects to be altered by this data collection method.

Future research might draw on this research to focus on assessing the impact of public campaigns on different indicators of stigma, so as to provide a more nuanced picture of how it operates and can be changed, enabling campaigns to target its various components. Indeed, despite good intentions to reduce the stigma associated with loneliness, campaigns and other media discussions around loneliness can make stigma worse because they often describe loneliness as something that is purely negative and must be eliminated (‘loneliness is the leprosy of the 21st century’, Ferguson, 2018; ‘Loneliness: Contagious like a bad cold’, Daily Express, 2009). Those designing future campaigns or interventions are encouraged to think about the various ways in which stigma is transmitted and experienced, so as to more deliberately and appropriately focus on its manifestations.

### Conclusion

This study reveals several ways in which the stigma of loneliness is manifested and how it varies by age, gender and the extent to which the country where people live is more or less individualistic. Stigma-related perceptions were stronger among young people, men and those living in collectivist societies. However, they differed slightly by indicator and were revealed in all groups – men, women, young, old, individualistic or collectivist – highlighting that it is less crucial to identify who stigmatizes or feels stigmatized, and more important to understand how this happens and how it might be addressed. We believe these findings will pave the way for a better understanding of the stigma associated with loneliness, so as to enable better and more efficacious interventions.

## Supplemental Material

sj-pdf-1-spr-10.1177_02654075221087190 – Supplemental Material for Exploring the nature and variation of the stigma associated with loneliness Click here for additional data file. Supplemental Material, sj-pdf-1-spr-10.1177_02654075221087190 for Exploring the nature and variation of the stigma associated with loneliness by Manuela Barreto, Jolien van Breen, Christina Victor, Claudia Hammond, Alice Eccles, Matthew T Richins and Pamela Qualter in Journal of Social and Personal Relationships

## Note

1. See Supplementary Materials for the results when participants’ own feelings of loneliness are not included as a covariate.
