# Supplementary material for: Exploring the nature and variation of the stigma associated with loneliness
Source: J Soc Pers Relat. 2022 Apr 21;39(9):2658–79. doi: 10.1177/02654075221087190 (PMC9386761; doi:10.1177/02654075221087190)
Supplement: sj-pdf-1-spr-10.1177_02654075221087190 – Supplemental Material for Exploring the nature and variation of the stigma associated with loneliness [file sj-pdf-1-spr-10.1177_02654075221087190.pdf]

Supplementary Files

Sample selection details

A total of 48,550 people completed the BBC Loneliness Experiment. The analyses reported in the current paper include gender, age, and country-level individualism as between-participant predictors. Therefore, those participants who did not provide information on these variables are not included in these analyses. Specifically, 762 participants did not provide their age, and we also excluded two participants who were under 16 years old. For country-level individualism, each participant was assigned a score on the Hofstede's Individualism Index based on their country of residence (1997, updated 2015), which provides cultural individualism scores for 101 countries, on scale ranging from 6 (Guatemala) to 91 (United States), with higher scores representing greater country-level individualism. Out of our total sample, 2013 participants could not be classified because their country of residence did not appear in the Hofstede database. Further, only those who described their gender as female or male were included in the analyses (N = 48,207), since we did not have sufficient data to perform a meaningful analysis of participants who reported their gender to be ‘other’ (N=235) or who preferred not to indicate their gender (N=108).

Wording of all questions on Qualtrics questionnaire

Loneliness feelings. We used four items from the UCLA scale: “In general, do you feel a lack of companionship?”, “In general, do you feel left out?”, “In general, do you feel isolated from others?”, and “In general, do you feel in tune with people around you?”. Participants responded to these items three times, first reporting how frequent the feeling is (from 1 = never to 5 = always), how intense (from 1 = not intense at all to 5 = very intense), and how long it lasts (1 = hours, 2 = days, 3 = weeks, 4 = months, 5 = longer). These ratings are highly correlated and reveal similar effects of gender, age, and cultural individualism (see

Barreto et al., 2020). Therefore, and given that the original UCLA scale is rated on frequency, for this paper we used frequency ratings as a covariate.

Age. "How old are you", responses were open and specified in years.

Gender. "What is your gender?", response alternatives: Male, female, other, prefer not to say.

Individualism. "Which country are you currently living in?". To assess individualism, each participant was assigned a score on Hofstede's Individualism Index based on their country of residence (Hofstede, 1997) using the tool available on this webpage: https://www.hofstede-insights.com/product/compare-countries/

Impressions of people who are feeling lonely. Based on Lau and Gruen (1992): **"**For this next task, we would like you to imagine a person who is feeling lonely. Please tell us what you think about them using the adjective pairs below. Choose were you think the person would fall between each pair of words". Pairs were: relaxed-nervous; steady-shaky; refreshed-tired; stable-unstable; healthy-sick; happy-sad; satisfied-dissatisfied; nice-awful; kind-cruel; friendly-unfriendly; good-bad; attractive-ugly; smart-dumb; successful-unsuccessful; superior-inferior; sharp-dull; valuable-worthless; confident-unsure; strong-weak; active-passive; sincere-insincere. Ratings were made on 7 point scales, with higher values reflecting more positive ratings.

Causal attributions for loneliness. Based on the work of Michela, Peplau, and Weekes (1982): **"**For the following statements, please rate how much you estimate the person described in each statement is likely to feel lonely or not: The person believes there is little chance of making a new friendship." Response scale: from 1 = this person is not very lonely to 5 = this person is very lonely. The remaining items were: "The person is afraid of being rejected if he or she tries to state a friendship or relationship", "The person doesn't try hard enough to meet someone"; "The person hasn't' had any luck meeting people", "The person

doesn’t know what to do to start a friendship or a relationship”, “The person is shy”, “The person is physically unattractive”, “This person believes other people have their own groups of friends and aren’t interested in this person”; “This person believes other people are afraid of making friends”; “The person is always in impersonal situations with too many people”; “There aren’t enough opportunities to meet people”; “Other people don’t try to make friends”; “The person has an unpleasant personality”.

Controllability of loneliness. “If you think about when you feel lonely, to what extent do you agree or disagree that the feeling of loneliness is caused by something you can change?”, “If you think about when you feel lonely, to what extent do you agree or disagree that the feeling of loneliness is caused by something you can control?”, “If you think about when other people feel lonely, to what extent do you agree or disagree that the feeling of loneliness is caused by something they can change?”, and “If you think about when other people feel lonely, to what extent do you agree or disagree that the feeling of loneliness is caused by something they can control?”

Perceived stigma in the community. “Below, you will find statements about the community in which you live. Think about your community and say whether you agree or disagree with each statement”. Statements were: “In general, people in the community where I live think that people who are lonely are less worthy than others”; “In general, people in the community where I live respect people who are lonely”; “In general, people in the community where I live think there is something wrong with people who are lonely”; and “In general, people in the community where I live tend to think that being lonely is a sign of weakness”. Responses were provided on 7 point scales, with 1 = strongly disagree and 7 = strongly agree.

Shame surrounding loneliness. “Think about a time when you have felt lonely. How much do you agree or disagree with the following statements.” Statements were: “When I

feel lonely, I feel ashamed about it"; "When I feel lonely, I am too embarrassed to admit that to others"; and "When I feel lonely, I do not talk to others about this". Responses were provided on 7 point scales, from 1 = strongly disagree to 7 = strongly agree.

Concealing loneliness. "You have recently started working at a new workplace. One day during the lunch break, one of your colleagues talks about her cousin, who is lonely. She goes into some detail about her cousin's life. Your colleague then begins to talk more generally about people who are lonely. Your colleagues do not know that you are lonely. If you were to find yourself in this situation, having this conversation with your colleague, would you choose to reveal this fact about yourself or would you, instead, choose to conceal the fact that you are lonely?". Responses were provided on a 7 point scale, from 1 = would definitely reveal to 4 = unsure, to 7 = would definitely not reveal.

Supplementary Analyses

Results if feelings of loneliness are not included in the model. In the manuscript text, we include participants' own feelings of loneliness as a covariate when studying the stigma associated with loneliness. After all, it stands to reason that a person who feels lonely themselves may have a different view of the stigma of loneliness than a person who does not personally feel lonely. Here, we report the results when that covariate is not included, that is, when the model includes only the central predictors (gender, age, cultural individualism) and their interactions.

Impressions of people who feel lonely. None of the effects reached significance. The effect of country-level individualism, which had reached significance when the covariate is included (p=.002) now drops to non-significance (p=.057).

Causal Attributions for Loneliness. Whereas before there were no main effects, now the main effects of age β =0.10, $F(1,34310)$ = 217.16, p<.001 and gender β =0.15, $F(1,34310)$ =148.98, p<.001 reached significance. As before, there was evidence for an

interaction between gender and age, $F(1,34310)=29.36$, $p<.001$, and the 3-way interaction between gender, age and country-level individualism just reached significance as well, $F(1,34310)=8.08$, $p=.004$. Breakdown of these interactions showed the same patterns as before: younger women were the least likely of all groups to differentiate between internal and external attributions for loneliness, and this was especially true in more individualistic cultures. In highly individualistic cultures, young women indicate no preference for internal (vs external) attributions (M= - 0.02, SD= 0.72), this differentiates them from young men, $M_{diff}=.11$, $t(34310) = 10.20$, $p<.001$, and from older women, $\beta=.004$, $t(34310)=12.02$, $p<.001$. No other effects were significant with $p < .01$.

Controllability of Loneliness. With regard to the predictors, there was a main effect of gender, $M_{diff}=.09$, $F(1, 37182) = 88.64$, $p<.001$, and country-level individualism, $\beta= - .08$, $F(1, 37182) = 180.28$, $p<.001$. The main effect of gender showed that men found loneliness more controllable than women. The main effect of country-level individualism showed that in more individualistic cultures, people find loneliness *less* controllable. The interaction between those two terms (which reached significance before) now dropped to non-significance, $F(1, 37182) =3.06$, $p=.080$. No other effects were significant with $p < .01$.

Perceived Stigma in the community. There were main effects of the three predictors on perceived stigma in the community: Gender, $M_{diff} = .19$, $F(1, 7834)=78.48$, $p<.001$, age, $\beta= - .17$, $F(1, 7834)= 335.60$, $p<.001$, and individualism, $\beta= -.10$, $F(1, 7834)=59.33$, $p<.001$. Older people, women, and those in more individualistic cultures perceived *less* stigma in the community relative to younger people, men, and those in collectivistic cultures. No other effects were significant with $p < .01$.

Shame surrounding loneliness. Shame surrounding loneliness was predicted by main effects of all other predictors, gender $M_{diff}= -.10$, $F(1,8190)=7.32$, $p=.007$, age, $\beta = - .16$, $F(1, 8190)=354.37$, $p<.001$, and country-level individualism, $\beta = .10$, $F(1, 8190)=97.97$, $p<.001$.

These main effects show that shame was higher amongst women, younger people, and those in individualistic cultures, relative to men, older people, and those in collectivistic cultures. The interaction between gender and individualism, which reached significance before, now dropped to non-significance, F=1.34, p=.246. No other effects were significant with $p < .01$.

Inclination to conceal loneliness. The inclination to conceal loneliness was predicted by main effects of gender, β = -.05, $F(1, 8793)$= 7.87, p= .005, age, β = -.19, $F(1, 8793)$=329.87, p<.001, and country-level individualism, β = .10, $F(1, 8793)$=80.87, p<.001. The inclination to conceal feelings of loneliness was stronger amongst younger people, women, and those in more individualistic cultures, relative to older people, men, and those in more collectivistic cultures. No other effects were significant with $p < .01$.

Analysis using separate impression categories. In the manuscript we take together all impression items into a single scale. However, the original authors (Lau and Gruen, 1992) differentiate four impression categories: Sociability, Adjustment, Competence and General Evaluation. In this section we offer the analysis separated by the different impression categories.

Sociability. Sociability impressions of people who feel lonely were predicted by one's own feelings of loneliness, β = -0.06 , $F(1, 8793)$= 43.48, p< .001, so that those who feel more lonely themselves report less negative impressions of the sociability of people who feel lonely compared to those who feel less lonely themselves. Additionally, there was a main effect of age, β = 0.06 , $F(1, 8793)$= 31.68, p< .001, so that older people reported more negative impressions of the sociability of people who feel lonely compared to younger people. No other effects were significant with $p < .01$.

Adjustment. Adjustment impressions were affected by main effects of all four predictors, but no interactions. People who felt more lonely themselves, rated people who feel lonely as *less* well-adjusted than did those who do not feel lonely themselves, β = 0.04, F(1,

8793)= 16.006, $p < .001$. Women rated people who feel lonely as *less* well-adjusted than did men, $M_{diff} = -0.13$, $F(1, 8793) = 33.42$, $p < .001$. Older people rated people who feel lonely as better adjusted than did younger people, β = -0.08 , $F(1, 8793) = 62.24$, $p < .001$. Finally, those in more individualistic cultures, rated people who feel lonely as less well-adjusted than did those in more collectivistic cultures, β = 0.04, $F(1, 8793) = 32.22$, $p < .001$. No other effects were significant with $p < .01$.

Competence. Competence impressions were affected by main effects of one's own feelings of loneliness, gender, and country-level individualism. People who felt more lonely themselves, rated people who feel lonely as *less* competent than did those who do not feel lonely themselves, β = 0.06, $F(1, 8909) = 27.56$, $p < .001$. Older people rated people who feel lonely as less competent than did younger people, β = 0.07 , $F(1, 8909) = 34.68$, $p < .001$. Finally, those in more individualistic cultures rated people who feel lonely as less well-adjusted than did those in more collectivistic cultures, β = 0.03, $F(1, 8909) = 17.08$, $p < .001$. No other effects were significant with $p < .01$.

General Evaluation. Finally, for general evaluation of people who feel lonely, none of the terms reached significance with $p < .01$.

Taken together, the results on these different impression categories seem somewhat scattered. For instance, there are considerable differences between the effects that appear for ratings of sociability and ratings of adjustment: the effects of age are reversed and so are the effects of one's own feelings of loneliness. The effect of country-level individualism appeared (in the same direction) both for the Adjustment and Competence impressions, and indeed it is this effect that seems most robust, appearing also when taking together all these items into a single scale.

Tables showing the full regression model for all variables

The tables below (A-F) show the full multilevel regression model for each of the central DVs.

Table A. Full regression model for the Impressions measure

					95% CI	
**Term**	**Estimate**	***SE***	**F-value**	**p-value**	**Lower bound**	**Upper bound**
Gender [-1 = men; 1 = women]	0.01	0.02	0.71	0.398	-0.02	0.05
Age	0.00	0.01	0.29	0.589	-0.03	0.03
Country-level Individualism	0.03	0.02	1.78	0.196	0.00	0.06
OwnLoneliness	0.01	0.01	2.26	0.133	0.00	0.03
Gender x Age	0.01	0.02	0.61	0.437	-0.02	0.05
Gender x Individualism	-0.03	0.02	2.62	0.105	-0.06	0.01
Age x Individualism	0.01	0.01	1.19	0.276	-0.01	0.03
Gender x Age x Individualism	0.00	0.02	0.04	0.840	-0.03	0.03

Random Effect	Estimate	SE	df	Likelihood ratio test	p-value
Country Residence (intercept)	0.01	0.09	1.00	21.688	0.000
Residual	0.50	0.71			

Table B. Full regression model for the Causal Attributions (difference score – see main text).

					95% CI	
**Term**	**Estimate**	***SE***	**F-value**	**p-value**	**Lower bound**	**Upper bound**
Gender [-1 = men; 1 = women]	-0.07	0.01	114.69	0.000	-0.08	-0.05
Age	0.03	0.01	169.93	0.000	0.02	0.04
Country-level Individualism	0.00	0.01	0.00	0.987	-0.02	0.01
OwnLoneliness	0.04	0.00	206.78	0.000	0.04	0.05
Gender x Age	0.03	0.01	23.82	0.000	0.02	0.04
Gender x Individualism	0.01	0.01	1.80	0.180	0.00	0.02
Age x Individualism	-0.01	0.00	0.00	0.977	-0.02	0.00
Gender x Age x Individualism	0.02	0.01	6.44	0.011	0.00	0.03

Random Effect	Estimate	SE	df	Likelihood ratio test	p-value
Country Residence (intercept)	0.00	0.03	1.00	21.69	0.000
Residual	0.25	0.50			

Table C. Full regression model for the measure of Controllability

Term	Estimate	*SE*	F-value	p-value	95% CI Lower bound	Upper bound
Gender [-1 = men; 1 = women]	-0.08	0.01	86.39	0.000	-0.09	-0.06
Age	-0.03	0.01	16.23	0.000	-0.04	-0.01
Country-level Individualism	-0.02	0.01	14.32	0.000	-0.04	-0.01
OwnLoneliness	-0.20	0.00	2839.39	0.000	-0.21	-0.20
Gender x Age	0.02	0.01	5.37	0.020	0.00	0.04
Gender x Individualism	-0.02	0.01	2.99	0.084	-0.03	0.00
Age x Individualism	-0.01	0.01	0.79	0.373	-0.02	0.00
Gender x Age x Individualism	0.01	0.01	0.51	0.477	-0.01	0.02

Random Effect	Estimate	SE	df	Likelihood ratio test	p-value
Country Residence (intercept)	0.00	0.07	1.00	303.79	0.000
Residual	0.53	0.73			

Table D. Full regression model for the measure of community stigma.

Term	Estimate	*SE*	F-value	p-value	95% CI Lower bound	Upper bound
Gender [-1 = men; 1 = women]	-0.15	0.03	28.22	0.000	-0.21	-0.10
Age	-0.18	0.02	161.94	0.000	-0.23	-0.14
Country-level Individualism	-0.09	0.03	16.64	0.000	-0.15	-0.03
OwnLoneliness	0.37	0.01	773.78	0.000	0.34	0.39
Gender x Age	-0.01	0.03	0.10	0.749	-0.07	0.05
Gender x Individualism	-0.04	0.03	2.28	0.131	-0.10	0.01
Age x Individualism	0.00	0.02	0.22	0.637	-0.03	0.04
Gender x Age x Individualism	0.01	0.03	0.10	0.748	-0.04	0.06

Random Effect	Estimate	SE	df	Likelihood ratio test	p-value
CountryResidence (intercept)	0.05	0.22	1.00	47.68	0.000
Residual	1.36	1.17			

Table E. Full regression model for Shame surrounding loneliness.

Term	Estimate	*SE*	F-value	p-value	95% CI Lower bound	Upper bound
Gender [-1 = men; 1 = women]	0.13	0.04	13.27	0.000	0.06	0.20

Age	-0.29	0.03	226.24	0.000	-0.34	-0.23
Country-level Individualism	0.02	0.03	4.50	0.039	-0.05	0.08
OwnLoneliness	0.60	0.02	1362.53	0.000	0.57	0.63
Gender x Age	0.02	0.04	0.25	0.616	-0.05	0.09
Gender x Individualism	0.08	0.04	4.69	0.030	0.01	0.16
Age x Individualism	-0.04	0.02	3.39	0.065	-0.09	0.01
Gender x Age x Individualism	0.02	0.04	0.25	0.616	-0.05	0.09

Random Effect	Estimate	SE	df	Likelihood ratio test	p-value
Country Residence (intercept)	0.02	0.16	1.00	47.31	0.000
Residual	2.17	1.47			

Table F. Full regression model for Intention to Conceal loneliness

					95% CI	
**Term**	**Estimate**	***SE***	**F-value**	**p-value**	**Lower bound**	**Upper bound**
Gender [-1 = men; 1 = women]	0.01	0.04	0.02	0.878	-0.07	0.08
Age	-0.19	0.03	66.28	0.000	-0.25	-0.12
Country-level Individualism	0.02	0.03	1.25	0.289	-0.04	0.08
OwnLoneliness	0.27	0.02	236.76	0.000	0.24	0.31
Gender x Age	0.05	0.04	1.56	0.212	-0.03	0.13
Gender x Individualism	0.01	0.04	0.12	0.729	-0.07	0.09
Age x Individualism	-0.05	0.03	5.53	0.019	-0.10	0.00
Gender x Age x Individualism	0.02	0.04	0.38	0.539	-0.05	0.09

Random Effect	Estimate	SE	df	Likelihood ratio test	p-value
Country Residence (intercept)	0.01	0.09	1.00	0.366	0.545
Residual	2.78	1.67			

Table G. Number of Participants and Hofstede Index per Country of Residence

Full Sample

Country of Residence	Hofstede Individualism	N observations
Guatemala	6	5
Ecuador	8	12
Panama	11	16
Venezuela	12	5
Colombia	13	30
Pakistan	14	48
Indonesia	14	63

Costa Rica	15	20
Peru	16	13
Trinidad and Tobago	16	37
Taiwan	17	36
Korea (South)	18	25
El Salvador	19	2
Bangladesh	20	20
Vietnam	20	23
Thailand	20	70
China	20	98
Singapore	20	184
Chile	23	29
Serbia	25	20
Hong Kong	25	105
Malaysia	26	90
Slovenia	27	16
Portugal	27	76
Bulgaria	30	40
Mexico	30	68
Romania	30	81
Philippines	32	73
Croatia	33	23
Greece	35	94
Uruguay	36	5
Turkey	37	153
Brazil	38	71
Jamaica	39	20
Russia	39	155
Iran	41	7
Morocco	46	7
Argentina	46	53
Japan	46	99
India	48	282
Spain	51	1
Slovakia	52	14
Israel	54	59
Austria	55	60
Czech Republic	58	58
Malta	59	40
Estonia	60	10
Lithuania	60	20
Luxembourg	60	22
Poland	60	104
Finland	63	61
Germany	67	552
Switzerland	68	223

Norway	69	136
Latvia	70	13
Ireland	70	507
Sweden	71	169
France	71	589
Denmark	74	95
Belgium	75	147
Italy	76	210
New Zealand	79	324
Hungary	80	37
Netherlands	80	253
Canada	80	1157
Great Britain	89	33304
Australia	90	992
United States	91	4117
		45548

'Stigma' branch

Country of Residence	Hofstede Individualism	N observations
Guatemala	6	1
Ecuador	8	2
Panama	11	1
Venezuela	12	2
Colombia	13	7
Indonesia	14	11
Pakistan	14	6
Costa Rica	15	7
Peru	16	2
Trinidad and Tobago	16	4
Taiwan	17	8
Korea (South)	18	5
Bangladesh	20	2
China	20	18
Singapore	20	43
Thailand	20	17
Vietnam	20	4
Chile	23	11
Hong Kong	25	30
Serbia	25	5
Malaysia	26	23
Portugal	27	12
Slovenia	27	2
Bulgaria	30	9
Mexico	30	19

Romania	30	16
Philippines	32	20
Croatia	33	2
Greece	35	20
Uruguay	36	3
Turkey	37	28
Brazil	38	15
Jamaica	39	3
Russia	39	30
Iran	41	2
Argentina	46	10
Japan	46	22
Morocco	46	2
India	48	39
Slovakia	52	4
Israel	54	9
Austria	55	10
Czech Republic	58	8
Malta	59	6
Estonia	60	2
Lithuania	60	4
Luxembourg	60	4
Poland	60	20
Finland	63	8
Germany	67	116
Switzerland	68	51
Norway	69	28
Ireland	70	101
Latvia	70	2
France	71	122
Sweden	71	43
Denmark	74	28
Belgium	75	25
Italy	76	38
New Zealand	79	69
Canada	80	243
Hungary	80	8
Netherlands	80	55
Great Britain	89	7021
Australia	90	218
United States	91	848
